# Prevalence of Antipsychotic Polypharmacy and Associated Factors among Outpatients with Schizophrenia Attending Amanuel Mental Specialized Hospital, Addis Ababa, Ethiopia

**DOI:** 10.1155/2016/6191074

**Published:** 2016-01-24

**Authors:** Siranesh Tesfaye, Nigussie Debencho, Teresa Kisi, Minale Tareke

**Affiliations:** ^1^Department of Psychiatry, Felege Hiwot Referral Hospital, Bahir Dar, Ethiopia; ^2^Department of Pharmacology, Amanuel Mental Specialized Hospital, Addis Ababa, Ethiopia; ^3^Department of Epidemiology and Biostatistics, Institute of Public Health, College of Medicine and Health Science, University of Gondar, Gondar, Ethiopia; ^4^Bahir Dar University, College of Medicine and Health Science, Bahir Dar, Ethiopia

## Abstract

*Background.* Despite recommendations by guidelines to avoid combinations of antipsychotics unless after multiple trials of antipsychotic monotherapy, it is quite a common practice to use combinations. This practice leads to unnecessary expenses and exposes the patient to severe drug adverse effects.* Methods*. An institution based cross-sectional study was conducted from April to May 2014. Systematic random sampling technique was used to select 423 study subjects. Logistic regression analysis was conducted to identify associated factors of antipsychotic polypharmacy among schizophrenia outpatients.* Result*. The overall prevalence of antipsychotic polypharmacy was found to be 28.2%. Extra pyramidal side effects (AOR = 2.80; 95% CI: 1.38, 5.71), repeated psychiatric hospitalization (AOR = 2.83; 95% CI: 1.45, 5.50), history of substance use (AOR = 2.82; 95% CI: 1.36, 5.88), longer duration of treatment (AOR = 2.10; 95% CI: 1.14, 3.87), and drug nonadherence (AOR = 1.84; 95% CI: 1.14, 2.98) were found to be significantly associated with antipsychotic polypharmacy.* Conclusion*. Prevalence of antipsychotic polypharmacy was found to be high among the current study participants. Individuals who had extra pyramidal side effects, admission, substance use, duration of treatment, and drug nonadherence were associated with antipsychotic polypharmacy.

## 1. Introduction

Schizophrenia is a severe mental illness with a serious impact on the lives of patients and their families. Treatment with antipsychotic drugs has been the main part of treatment and management of psychotic patients globally [1]. These patients exhibit marked interindividual variability in their response to antipsychotics and some of them have poor response to several antipsychotics or even no response at all. Therefore, common strategy in management of these difficult to treat patients is combination of antipsychotic agents [2].

Antipsychotic polypharmacy (APP) is the use of two or more antipsychotic drugs at a time for a given patient. Study in USA reported a prevalence of 27.5% antipsychotic combination which is similar to that in South Africa (28.6%) and the study done in six East Asian countries and territories (China, Hong Kong, Japan, Korea, Singapore, and Taiwan) revealed that the prevalence of antipsychotic polypharmacy was 45.7% [3, 4], while a Nigerian study reported a 92% prevalence polypharmacy [5]. These discrepancies in prevalence of antipsychotic combination across studies may be accounted for by differences in the definition of antipsychotic combination and also availability and type of medical insurance for schizophrenia patients as well as clinical experience and knowledge of psychopharmacology by medical practitioners [6].

Despite recommendations by guidelines to avoid combinations of antipsychotics unless after multiple trials of antipsychotic monotherapy, it is quite a common practice to use combinations [7]. In addition to this, patients who need antipsychotic doses more than the maximum recommended are often prescribed two antipsychotics [3].

When more than one antipsychotic drug is prescribed at a time, it is difficult to identify the antipsychotic agent which reduced the psychotic symptoms and the other which is responsible for the adverse effects. The frequently reported reason for antipsychotic polypharmacy is that if one agent does not achieve the desired therapeutic outcome for controlling positive and/or negative symptoms, another medication within the same class can be added to address the uncontrolled symptoms. But this practice on the contrary leads to unnecessary expenses and exposes the patient to severe drug adverse effects.

Therefore, long term use of antipsychotic polypharmacy is recommended only as a last resort after having exhausted monotherapy alternatives [2, 8]. In this study, frequency of antipsychotic combinations in Amanuel Mental Specialized Hospital was examined. Furthermore, factors associated with antipsychotic combination were investigated.

## 2. Methods

A cross-sectional study design was conducted at Amanuel Mental Specialized Hospital (AMSH) in Addis Ababa from April to May, 2014. AMSH is one of the oldest hospitals established in 1937 and located in western part of Addis Ababa, the capital city of Ethiopia. The hospital is playing its pivotal role as a training institute for psychiatric professionals so as to expand psychiatry service to the primary health care system of the country. There were about 51,204 schizophrenia patients who had regular follow-up in a year period at outpatientdepartment; and on average 4,267 schizophrenia patients had monthly follow-up. The study population was schizophrenia outpatients who were on regular treatment and who had follow-up during the study period at AMSH. Patients aged 18 years and above and who had one or more previous visits were involved in the study. Patients with medical or neurological illnesses, who had no insight, and who were unable to communicate were excluded from the study.

### 2.1. Sample Size and Sampling Procedures

The sample size was calculated using the formula [*n* = ((*zα*/2)2*p*(1 − *p*))/*d*2] for estimating a single population proportion at 95% confidence interval (CI) (*Zα*/2 = 1.96) and 5% margin of error. Due to absence of data in the country, proportion of population who took polypharmacy antipsychotic among schizophrenia patients was assumed to be 50%, and by adding 10% contingency for nonresponse rate, a total of 423 study populations were involved.

The total number of schizophrenia patients who visit the hospital over the previous 6 months was taken from records and the average number per month calculated and it was found to be 4267. Systematic random sampling technique was used to select the study subjects. The sampling fraction is 4267/423 = 10. Hence, patient was selected every ten intervals. The first individual was selected by lottery method among the first ten patients who visited the hospital.

### 2.2. Data Collection and Quality Control

The data was collected using a pretested structured questionnaire developed in English and translated to Amharic and then to English by expertise and senior psychiatrist to ensure its consistency. The questionnaire was pretested on 5% of the sample size at St. Paul's Hospital one week before data collections. Both chart review and interview aided questionnaire were used to collect information from the study participants. Data was collected by three trained diploma psychiatry nurses and one supervisor (B.S. nurse) for a period of one month. Face-to-face interview was employed using local Amharic language.

Data regarding type of antipsychotics, duration of treatment, admission frequency, and duration of illness was filled from patient record. Drug nonadherence was assessed using the eight-item version of self-reporting questionnaire of Morisky medication adherence rating scale (MMARS); it is validated and is used as a tool in certain African countries including South Africa, Nigeria, and Kenya. The cut point of 3 and above is used to define nonadherence [9, 10] and the extra pyramidal side effects (EPS) were assessed using Simpson-Angus Scale (SAS); it is a 10-item rating scale that has been used widely for assessment of Neuroleptic Induced Parkinsonism (NIP) in both clinical practice and research settings. It consists of one item measuring gait (hypokinesia), six items measuring rigidity, and three items measuring glabella tap, tremor, and salivation, respectively. The cut-off value for screening NIP is 0.65 or more [11]. The use of two or more antipsychotic drugs at a time/simultaneously for a given patient for at least 30 days was taken as antipsychotic polypharmacy.

Two-day training was given to orient data collectors and supervisor on the questionnaire to be used, the purpose of the study, and how to approach respondents and obtain consent. The data collectors were supervised daily and the filled questionnaires were checked for completeness and consistency by supervisor and principal investigator.

### 2.3. Data Management and Analysis

Data was cleaned, edited, and entered using Epi info version 3.5 statistical software and then exported to SPSS version 20 for further analysis. Description of the collected data was done using frequency, percentages, means, and standard deviations. Logistic regression was performed to assess the association between binary outcomes and different explanatory variables. Bivariate analysis was first conducted for each potentially explanatory risk factor. Variables that satisfied *p* value <0.2 were selected for further analysis using multivariate logistic regression analysis in order to control confounding effects. The strength of association was interpreted using odds ratio (OR) and confidence interval (CI). *p* value <0.05 was considered statistically significant in this study.

### 2.4. Ethical Consideration

Ethical clearance was obtained from University of Gondar and AMSH. Informed consent was obtained from each respondent. They were given the right to refuse to take part in the study as well as to withdraw at any time during the interview process. Confidentiality was maintained throughout the study.

## 3. Results

### 3.1. Sociodemographic Characteristics and Other Related Factors

A total of 412 patients of schizophrenia were included in the study with a response rate of 97.4%. The mean age of the participants was 35.28 (±10.35 years), with age range of 18–85. The majority (69.7%) of participants were males, Orthodox Christians (65.5%), never married (65.5%), and from urban area (68.0%) and earn less than 750 birr monthly income (73.3%) (Table 1).

### 3.2. Clinical and Patient Related Factors

Among the participants, the duration of illness and duration of treatment above ten years were 38.6% and 28.9%, respectively. On the other hand, 13.6% of the participants had two or more previous psychiatric admissions, had good adherence (59.0%) to their antipsychotic treatment, and had extra pyramidal side effect (10.4%) (Table 2).

### 3.3. Factors Associated with Antipsychotic Polypharmacy

The overall prevalence of any antipsychotic polypharmacy was 28.2%; of these 27.9% were on two antipsychotics; that is, 23.3% were on FGA + FGA and 4.6% were on FGA + SGA (Table 2).

Bivariate logistic regression analyses were done for the relationship of sociodemographic variables, patient related variables, and treatment or medication related variables with antipsychotic polypharmacy. The result of bivariate analysis revealed that sex, ethnicity, place of residence, marital status, occupational status, duration of illness, duration of treatment, number of psychiatric hospitalization days, history of active substance use, extra pyramidal side effect, and drug adherence were found to be significantly associated with antipsychotic polypharmacy. However, by multivariate logistic regression only duration of treatment, number of hospitalization days, history of active substance use, extra pyramidal side effect, and drug nonadherence were found to be statistically significant. Accordingly, patients who were on treatment for more than ten years were found to be about two times more likely to be on antipsychotic polypharmacy as compared to those who are on treatment for less than five years (AOR = 2.24; 95% CI: 1.29, 3.89). Concerning hospitalization status of study participants, patients who had two or more previous admissions were found to be three times more likely to be on antipsychotic polypharmacy as compared to those who had no previous admission (AOR = 3.16, 95% CI: 1.68, 5.94).

Regarding substance use, patients who were using psychoactive substances after initiation of treatment were found to be three times more likely to be on APP than patients who had no history of substance use (AOR = 1.69, 95% CI: 1.06, 2.71). Patients who had extra pyramidal side effect (EPS) were about three times more likely to be on APP when compared to those who had no EPS during the study period (AOR = 2.76, 95% CI: 1.38, 5.53). Patients who were nonadherent to their treatment were found to be two times more likely to be on APP as compared to those who had good adherence to antipsychotic treatment (AOR = 1.96, 95% CI: 1.22, 3.15) (Table 3).

## 4. Discussion

This study has attempted to identify the prevalence of antipsychotic polypharmacy (APP) and associated factors among schizophrenia outpatients attending AMSH. The overall prevalence of APP was found to be 28.2%.

The prevalence of antipsychotic polypharmacy in the current study area was found to be similar with the studies in USA, South Africa, and Jordan which were 27.5%, 28.6%, and 24.7%, respectively [3, 4, 12], but lower than the findings in Singapore, France, and Egypt which were 71.7%, 37.7%, and 37.6%, respectively [1, 12, 13]. On the contrary the finding of APP in this study is relatively higher than reports in Bahrain which is 10.8% [14]. The possible reason for discrepancies in prevalence rates of antipsychotic polypharmacy among studies could be explained by differences in sociodemographic characteristics, population difference, and instrument used which involves clinical judgments. Besides these, certain studies had used different inclusion criteria; for instance, in USA participants were those who were on treatment for 60 days [15].

Among participants who were on antipsychotic polypharmacy in the current study, almost all (99.1%) were taking two antipsychotics; of those 82.7% were taking combination of typical antipsychotics which is in line with findings in France [13]. This might be because of availability and cost of typical (first generation) antipsychotic drugs. Regarding the associated factors, participants who had history of substance use since the initiation of treatment were three times more likely to be on APP. The possible reason could be that psychoactive substances decrease the effect of antipsychotic drugs and the patient may have poor adherence; these in turn lead the patient to be on APP.

There was a strong association between extra pyramidal side effect (EPS) and APP in this study; individuals who had EPS were about three times more likely to be on APP as compared to those who had no EPS. This is in line with the findings in USA which reports a high prevalence of antipsychotic polypharmacy cases among patients who had EPS [16]. The possible reason could be that patients treated for schizophrenia receive antipsychotic drugs more than the necessary total daily doses and also limited access of antipsychotic drugs (atypical antipsychotic drugs) with less extra pyramidal side effects.

Those who had history of repeated admission were about three times more likely to be on APP as compared to those who do not have admission. This is similar with findings in USA schizophrenia outpatients which report an increased likelihood of hospital admission among polypharmacy cases [17]. The possible reason might be use of psychoactive substances, having poor compliance to their treatment, or increased medication side effects and finally these all worsen the positive symptom of schizophrenia and increase the relapse rate so patient might be admitted repeatedly.

Participants who had poor adherence to their treatment were two times more likely to be on APP. These patients could have a higher likelihood of acute psychiatric hospitalization because of forgetting or being unable to take medications and increasing side effects of medications and finally this would suggest interventions on multiple antipsychotics when compared to those who had good adherence.

A statistically significant association was also found between long treatment duration and APP. The possible reason could be that these patients might be lost to follow-up after initiation of treatment, might have poor prognosis, and experience increased adverse effects as a result of excess antipsychotic exposure to control the aggravated psychotic symptoms.

The strength of this study is the first of its kind in Ethiopia that determined the prevalence and associated factors for antipsychotic polypharmacy among schizophrenia patients. However, our limitations include clinical data pertaining to illness severity which were difficult to assess using cross-sectional studies.

## 5. Conclusion

Prevalence of APP was found to be high among the current study participants. Individuals who had extra pyramidal side effects, repeated psychiatric admission, history of active substance use, longer duration of treatment, and drug nonadherence were found to have significant association with antipsychotic polypharmacy. Clinicians have to monitor patients for treatment adherence and development of side effects before proceeding to APP. Further cohort study is needed to test the potential benefits and risks of specific antipsychotic combination therapies.

## Figures and Tables

**Table 1:** Distribution of sociodemographic characteristics of schizophrenia outpatients attending AMSH, June 2014.

Variable		Frequency	Percentage (%)
Sex	Male	287	69.7
	Female	125	30.3

Age	≤25	71	17.2
	26–35	163	39.6
	≥36	178	43.2

Marital status	Single	270	65.5
	Married	80	19.4
	Divorced	32	7.8
	Widowed	6	1.5

Residence	Urban	280	68.0
	Rural	132	32.0

Religion	Orthodox	231	65.5
	Muslim	80	19.4
	Protestant	51	12.4
	Others	4	2.7

Ethnicity	Amhara	148	35.9
	Oromo	112	27.2
	Gurage	93	22.4
	Tigre	21	5.1
	Others	38	9.2

Educational status	Uneducated	49	11.9
	1–8 grades	126	30.6
	9–12 grades	157	38.1
	Diploma and above	80	19.4

Occupation	Employed	68	16.5
	Private business	70	17.0
	Daily laborer	37	9.0
	Jobless	196	47.6
	Student	20	4.9
	Housewife	21	5.1

Monthly income (ETB)	<750 birr	302	73.3
	750–1199 birr	47	11.4
	≥1200 birr	63	15.3

**Table 2:** Distribution of clinical and patient related factors among schizophrenia patients at AMSH, Addis Ababa, Ethiopia, June 2014 (*n* = 412).

Variable name		Frequency	Percent
Duration of illness	<5 years	143	34.7
	5–10 years	110	26.7
	>10 years	159	38.6

Antipsychotic polytherapy	Yes	116	28.2
	No	296	71.8

Duration of treatment	<5 years	198	48.1
	5–10 years	95	23.1
	>10 years	119	28.9

Type of antipsychotics	^*∗*^FGA	250	60.7
	^*∗*^SGA	46	11.2
	FGA + FGA	96	23.3
	FGA + SGA	19	4.6
	FGA + FGA + SGA	1	0.2

Number of admissions	None	290	70.4
	One	66	16.0
	≥two	56	13.6

EPS	Yes	43	10.4
	No	369	89.6

Drug adherence	Yes	243	59.0
	No	169	41.0

Substance use (alcohol, Khat, and tobacco)	No	250	60.7
	Yes	162	39.3

^**∗**^FGA: first generation antipsychotics. ^*∗*^SGA: second generation antipsychotics.

**Table 3:** Factors associated with antipsychotic polypharmacy among schizophrenia outpatients under follow-up at AMSH Addis Ababa, Ethiopia, June 2014 (*n* = 412).

Variables		Polypharmacy (%)		COR, 95% CI	AOR, 95% CI
		Yes	No		
Sex	Male	92	195	**1.99 (1.19, 3.30)**	1.16 (0.58, 2.31)
	Female	24	101	1.00	1.00

Ethnicity	Amhara	37	75	1.07 (0.48, 2.35)	1.17 (0.46, 2.94)
	Oromo	32	116	0.60 (0.27, 1.31)	0.74 (0.29, 1.85)
	Tigre	10	11	1.97 (0.65, 5.89)	1.54 (0.43, 5.52)
	Gurage	25	68	0.80 (0.35, 1.81)	0.87 (0.82, 2.74)
	Others	12	26	1.00	1.00

Residence	Urban	88	192	**1.70 (1.04, 2.77)**	1.50 (0.82, 2.74)
	Rural	28	104	1.00	1.00

Marital status	Married	15	65	1.00	1.00
	Single	81	189	**1.86 (1.00, 3.45)**	0.73 (0.37, 1.43)
	Separated	11	13	**3.67 (1.37, 9.76)**	2.11 (0.83, 5.35)
	Divorced	8	24	1.44 (0.54, 3.84)	0.80 (0.32, 2.02)
	Widowed	1	5	0.86 (0.09, 7.97)	0.64 (0.06, 5.97)

Occupational status	Employed	17	51	3.17 (0.66, 15.02)	2.60 (0.45, 14.93)
	Private business	18	52	3.29 (0.69, 15.53)	1.79 (0.30, 10.66)
	Daily laborer	11	26	4.02 (0.79, 20.28)	2.26 (0.35, 14.56)
	Jobless	66	130	**4.82 (1.09, 21.33)**	2.99 (0.52, 16.91)
	Student	2	18	1.06 (0.13, 8.31)	1.16 (0.11, 12.13)
	Housewife	2	19	1.00	1.00

Duration of illness	<5 years	28	115	1.00	1.00
	5–10 years	34	76	**1.83 (1.03, 3.27)**	0.72 (0.29, 1.75)
	>10 years	54	105	**2.11 (1.24, 3.58)**	0.43 (0.13,1.46)

Duration of treatment	<5 years	37	161	1.00	1.00
	5–10 years	35	60	2.53 (1.46, 4.39)	**1.93** (1.07,3.49)^*∗*^
	>10 years	44	75	**2.55 (1.52, 4.27)**	**2.24** (1.29,3.89)^*∗*^

Number of admissions	None	65	225	1.00	1.00
	One	20	46	1.51 (0.83, 2.72)	1.50 (0.80, 2.80)
	Two or more	31	25	**4.29 (2.36, 7.78)**	**3.16 **(1.68,5.94)^*∗*^

Substance use (Khat, alcohol, and tobacco)	No	54	196	1.00	1.00
	Yes	62	100	**2.25 (1.45, 3.48)**	**1.69 **(1.06,2.71)^*∗*^

Extra pyramidal side effect	No	94	275	1.00	1.00
	Yes	22	21	**3.07 (1.61, 5.82)**	**2.76 **(1.38,5.53)^*∗*^

Drug adherence	Yes	52	191	1.00	1.00
	No	64	105	**2.24 (1.44, 3.46)**	**1.96 **(1.22,3.15)^*∗*^

^*∗*^ *p* value is significant at *p* < 0.05.
